# Application of Aluminum Hydroxide for Improvement of Label-Free SERS Detection of Some Cephalosporin Antibiotics in Urine

**DOI:** 10.3390/bios9030091

**Published:** 2019-07-23

**Authors:** Natalia E. Markina, Alexey V. Markin

**Affiliations:** Institute of Chemistry, Saratov State University, Astrakhanskaya 83, 410012 Saratov, Russia

**Keywords:** surface-enhanced Raman spectroscopy, cefazolin, cefoperazone, cefotaxime, ceftriaxone, cefuroxime, silver nanoparticles, therapeutic drug monitoring, sample pretreatment

## Abstract

This report is dedicated to development of surface-enhanced Raman spectroscopy (SERS) based analysis protocol for detection of antibiotics in urine. The key step of the protocol is the pretreatment of urine before the detection to minimize background signal. The pretreatment includes extraction of intrinsic urine components using aluminum hydroxide gel (AHG) and further pH adjusting of the purified sample. The protocol was tested by detection of a single antibiotic in artificially spiked samples of real urine. Five antibiotics of cephalosporin class (cefazolin, cefoperazone, cefotaxime, ceftriaxone, and cefuroxime) were used for testing. SERS measurements were performed using a portable Raman spectrometer with 638 nm excitation wavelength and silver nanoparticles as SERS substrate. The calibration curves of four antibiotics (cefuroxime is the exception) cover the concentrations required for detection in patient’s urine during therapy (25/100‒500 μg/mL). Random error of the analysis (RSD < 20%) and limits of quantification (20‒90 μg/mL) for these antibiotics demonstrate the applicability of the protocol for reliable quantitative detection during therapeutic drug monitoring. The detection of cefuroxime using the protocol is not sensitive enough, allowing only for qualitative detection. Additionally, time stability and batch-to-batch reproducibility of AHG were studied and negative influence of the pretreatment protocol and its limitations were estimated and discussed.

## 1. Introduction

Surface-enhanced Raman spectroscopy (SERS) has been proposed for therapeutic drug monitoring (TDM) because the analysis procedures are fast and simple, and the equipment is portable and has moderate cost [1]. Detection of antibiotics in blood and urine [2] is one of the main tasks of TDM, especially for people with renal function impairment [3,4]. The failure of renal functions causes the accumulation of the antibiotics in organism leading to enhancement their side effects.

Two types of SERS analysis have been proposed: label-based and label-free. Label-based analysis implies application of SERS-active plasmonic nanoparticles coated by Raman reporter (a compound with well-known and intensive Raman spectrum) and recognition layer (antibody, aptamer, etc.) [5]. This method possesses the maximal selectivity, but complexity of the structure and fabrication of SERS labels restricts their widespread application. Additionally, the high specificity leads to demand for the fabrication of a new label in case of changing of the target analyte. Label-free SERS detection is based on registration of enhanced Raman spectra of the analyte which is directly adsorbed onto the surface of SERS substrates [6]. In contrast to label-based SERS analysis, this method suffers from possible competitive interactions onto the surface of SERS substrate between the analyte and admixtures (e.g., other drugs or intrinsic components of biofluids). However, the advantages of this detection type include identification of unknown analyte(s), detection of structurally related analytes, and simplicity and cost efficiency of the SERS substrate preparation.

The improvement of reliability of label-free SERS detection can be achieved using sample pretreatment of complex objects before the analysis. Different approaches were proposed such as follows: (i) extraction of a target analyte from the sample [7,8,9]; (ii) masking of interfering components by suppression their SERS signal [10]; (iii) analyte purification approach based on removing interfering admixtures from the mixture (intrinsic and artificial urine components) [11,12].

In addition to competitive interactions, another challenge is the applicability of SERS protocol for the detection of structurally related compounds, e.g., antibiotics of the same class in urine. Indeed, most of the reported studies discuss the protocols which were developed and tested using only one representative of the class of drugs [1,7,8,9,10,12]. The difficulties with different antibiotic derivatives occur because new moieties may significantly change (i) adsorption of the analyte on the surface of SERS substrate; and (ii) the profile of the analyte SERS spectrum. In the case of sample pretreatment, different derivatives may also have different responses to the pretreatment. Thus, the developments of SERS based analysis protocols universal enough for detection of different representatives of the antibiotics within one class are in demand.

The aim of this study is the development of a sample pretreatment protocol based on the removal of interfering components (analyte purification approach) to improve SERS detection of antibiotics in the urine. The monitoring of the dynamic of antibiotic concentration in urine allows for control of antibiotic release from the body and indirect determination of its concentration in blood. Cephalosporin antibiotics cefazolin (CZL), cefoperazone (CPR), cefotaxime (CTX), ceftriaxone (CTR), and cefuroxime (CRX) were selected as the targets of interest due to their wide usage and adverse effects in case of uncontrolled therapeutic treatment [13]. Aluminum hydroxide gel (AHG) was proposed as the sorbent for analyte purification. Hydroxylamine stabilized silver nanoparticles (AgNPs) were used as SERS substrate due to their simple preparation and, more importantly, negligible contribution to background signal.

## 2. Materials and Methods

### 2.1. Materials

Silver nitrate and creatinine were purchased from Sigma-Aldrich. Aluminum sulfate (Al_2_(SO_4_)_3_·18H_2_O), anhydrous sodium carbonate, sodium hydroxide, hydrochloric acid (35%), sodium chloride, urea, hydroxylamine hydrochloride, and isopropyl alcohol (IPA) were purchased from Vekton Ltd. (Saint Petersburg, Russia). Powdered antibiotics used for injections were used as analytes during the study (Sintez JSC, Kurgan, Russia) (the structures are provided in Table S1; Supplementary Material). Bi-distilled water was used for preparation of the SERS substrate (AgNPs); distilled water was used in all other cases.

### 2.2. Preparation of SERS Substrate

Hydroxylamine stabilized AgNPs were prepared according to Leopold and Lendl’s protocol [14]. The solution of AgNO_3_ (0.1 mL, 0.5 M) was added to water (10 mL). The solutions of NH_2_OH·HCl (0.5 mL, 0.15 M) and NaOH (0.15 mL, 1.0 M) were added to water (40 mL). Then the diluted AgNO_3_ solution was added fast to the mixture of NH_2_OH·HCl and NaOH under hand-shaking. The final, grey–brown solution of AgNPs was stored at room temperature in tightly closed glass vial and used within one week.

### 2.3. Preparation of Aluminum Hydroxide Gel (AHG)

The synthesis of AHG is based on the modified protocol of Yurova et al. [15]. The samples were synthesized by simultaneous addition of aluminum sulfate (2.5 mL, 200 mg/mL) and sodium carbonate (2 mL, 120 mg/mL) aqueous solutions to water (9.5 mL) under vigorous stirring (500 RPM; RCT basic, IKA, Staufen, Germany); 0.5 mL portions of the reagents were used for addition. The mixture was kept for stirring for 5 min after addition of the last portion of aluminum salt. Then the mixture was placed to eight plastic tubes for microcentrifugation (1.75 mL of the mixture in each tube), centrifuged (1700 g, 2 min), and rinsed two times with water (2 mL each time). Finally, after rinsing steps, 1.5 mL of water was removed from each plastic tube and remained portions of AHG precipitate (~0.5 mL) were stored at ambient conditions and further used for pretreatment of urine.

### 2.4. Urine Collection and Sample Pretreatment

Fresh samples of human urine were collected from five healthy volunteers. All participants were provided with the complete description of the study before providing urine samples. Then the samples were anonymized and randomized. The study was not aiming to investigate functions/diseases of the human body or as a process of medical treatment. The antibiotics were used only for in vitro artificial spiking of collected urine samples and the participants did not administrate them. Therefore, approving the study by ethical committee and collecting informed consents were not performed.

The collection of the samples was performed during the day-time, i.e., the first urine after awaking was not used for the analysis. The samples were stored at 4 °C and used for analysis within one day after collecting. The samples were homogeneous before and after pretreatment; thus, no additional purification of the samples (filtration or centrifugation) was applied before analysis. Artificial spiking before analysis was used to contaminate the samples with the analyte of interest. In order to keep sample dilution constant, the spiking was performed by addition of 10 μL of the analyte stock solution (aqueous) to 990 μL of the urine. Blank urine samples were used as a reference.

**Figure 1:**
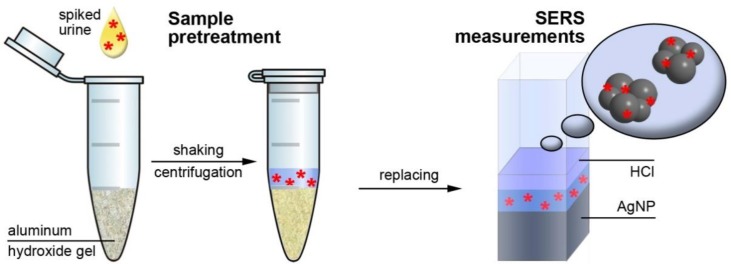
Scheme of urine sample pretreatment using aluminum hydroxide gel and surface-enhanced Raman spectroscopy (SERS) measurements.

The scheme of the urine pretreatment procedure is shown in Figure 1. A portion of blank or spiked urine (100 μL) was added to the plastic tube with the portion of AHG. The mixture was shaken for 5 min and centrifuged at 1700 g for 2 min. Then a portion of supernatant solution (100 μL for CZL, CPR, and CTX; 175 μL for CTR and CRX) was mixed with HCl (25 μL, 1 M) and AgNPs (450 μL) solutions and this mixture was used for SERS analysis. The averaged SERS signal for each analyte concentration was obtained using five different urine samples to account for an influence of matrix composition deviation.

Absorbance measurements in UV-visible range (spectrophotometer UV-1800, Shimadzu, Kyoto, Japan) were used to study sorption properties of AHG. A portion of analyzed solution (100 μL) was added to the plastic tube with the portion of AHG. The mixture was shaken for 5 min and centrifuged at 1700 *g* for 2 min. Then a portion of supernatant solution (150 μL) was used for absorbance measurements; quartz cuvette for a small sample volume (300 μL; 1 cm optical path length) was used.

### 2.5. SERS Measurements

A high-performance modular Raman system (Ocean Optics, Largo, FL, USA) was used for collecting SERS spectra; an itemized description of the system is provided in the Supplementary Material (Table S2). All spectra were measured in quartz cuvette (1 cm optical path length) with 1 s signal acquisition time; the intensity was measured in counts per second (cps). The SERS signals for blank urine and main intrinsic urine components were obtained by averaging 40 spectra; for spiked samples the averaging was performed using 50 spectra. The verification of the spectrometer adjustment was performed before measurements using IPA; additionally, the IPA band at 828 cm^−1^ was used to normalize the intensity of all SERS spectra.

## 3. Results and Discussion

### 3.1. SERS Signal of Pure Urine

**Figure 2:**
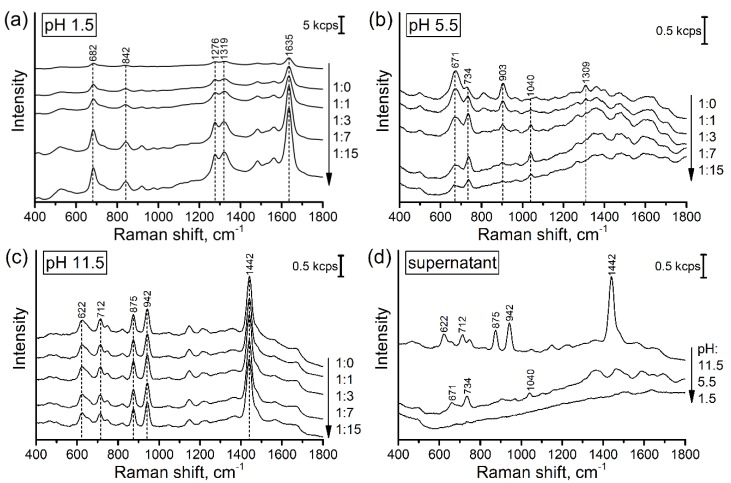
(**a**–**c**) The dependence of SERS spectra profiles of the pure urine samples on pH adjusting and dilution; the numbers near the arrows display volume ratios of urine to water. Important: the Y scale for graph (**a**) is 10-fold larger than for graphs (**b**,**c**). (**d**) An influence of pH value on SERS spectra of undiluted urine after its pretreatment with AHG. Three different urine samples were used to get averaged results.

Because SERS measurements are quite dependent on the spectrometer (optical system and excitation source), a reference investigation of the influence of pH adjusting and dilution on the intensity and profile of SERS spectra of the pure urine was performed (Figure 2). The trial experiments with artificial urine showed that it cannot satisfactorily reproduce the SERS signal and its deviation from real urine samples. Therefore, only real urine samples were used during the study to develop reliable analysis protocol.

#### 3.1.1. Influence of pH

The intensive SERS signal was observed at all tested pH values (Figure 2a–c). Undiluted urine has SERS signals of almost similar intensity at acidic and alkaline pH values and two times weaker signal in the neutral media. Changes of pH value significantly change the profile of SERS spectra because (de)protonation leads to altering sorption mechanism and activity of Raman bands. Control measurements of some pure intrinsic urine components (urea and creatinine, Figure S1) showed that creatinine is the main background forming compound in the case of alkaline media (Figure 2c). Urea does not contribute to the background, despite its excessive concentration in urine (21 mg/mL [16]). SERS bands observed in the acidic media can be attributed to urobilin and urobilinogen as by-product of bilirubin reduction [17]. Due to the presence of pyrrole subunits, these compounds have high Raman activity and affinity to the surface of silver-based SERS substrates leading to the formation of the intensive SERS signal.

#### 3.1.2. Influence of Dilution

The study of urine dilution showed that the used dilutions (2‒16 times) do not reduce SERS signal of the urine components. The dilution also does not influence the profiles of the spectra. These facts can mean excessive metabolite concentrations relative to the surface of SERS substrate and the strong interaction between the metabolites and the surface. Moreover, in the case of acidic pH, the dilution leads to almost 10-fold growth of SERS signal (Figure 2a) and the appearance of relatively intensive continuous featureless background (Figure S2).

The low SERS signal of undiluted acidified urine can be explained by formation of the intermolecular complexes between partially protonated urine components. The examples for such complexes were reported for creatinine [18] and uric acid [19], which tend to form stable di-, tri-, and tetramers. Because lone pairs of nitrogen atoms of these compounds are participated to the intermolecular bonding, the capability for interaction with SERS substrate of the complexes is reduced compared to separate molecules. Although there are no reports regarding the possible urobilin complexes, the richness of this compound by nitrogen atoms and relative flexibility of the structure lead to possibility for formation of inter- and intramolecular complexes with different affinity to the surface of SERS substrate. Thus, the growth of SERS intensity with dilution can be attributed to the collapsing of the complexes and increasing the interaction of the constituents with SERS substrate.

According to the results of Masilamani et al. [20], the native urine has fluorescence bands at 630 and 690 nm (λex = 400 nm) corresponded to porphyrin derivatives in neutral and protonated forms, respectively. Leiner at al. [21] also reported the presence of two fluorescence bands within the same region which experience significant growth of intensity in acidic media. Thus, although the wavelength of excitation source used for SERS generation (638 nm) is far from optimal (400 nm), its high intensity leads to possibility for excitation of fluorescence background (Figure S2). As in the case of SERS signal, the low fluorescence signal for undiluted urine can be associated with formation of intermolecular complexes which is responsible for concentration dependent self-quenching [22].

#### 3.1.3. Treatment by AHG

Summing-up the results described above, we can conclude that only pH adjusting and dilution cannot eliminate the background SERS signal formed by intrinsic urine components. This issue restricts the reliability of direct SERS based detection of antibacterial drugs in urine, particularly at low analyte concentrations. To overcome this challenge, we proposed sample pretreatment step in order to purify the analytes by extracting the main uric components from the urine. The extraction was performed using AHG and native urine. The SERS measurements of the supernatant solutions show that the SERS signal of acidified supernatant has the minimal intensity without prominent Raman bands (Figure 2d). The mechanism of purification can be explained by the interaction between electron lone pairs of the nitrogen containing components of urine (creatinine and urobilin) and positively charged aluminum ions which work as Lewis acid. This mechanism was earlier described and used for fabrication of aluminum doped molecularly imprinted silica gel with high specificity to creatinine [23]. In the case of small metabolites like creatinine (113 g/mol), the size exclusion extraction mechanism can also contribute because the antibiotics have significantly larger molar mass (424‒646 g/mol, Table S1) and poorly penetrate inside AHG. Although this mechanism does not enable complete extraction of some metabolites (e.g., creatinine in alkaline media, Figure 2c), it has enough efficiency to minimize background signal in acidic media. Therefore, the purification and acidification of urine samples enable us to minimize SERS signal background leading to improving reliability of the antibiotic detection.

### 3.2. Stability of AHG during the Storage and Batch-to-Batch Reproducibility

Besides the advantage of background reduction, the addition of a sample pretreatment step can also influence analysis error. For example, AHG is a gel and it can experience composition changes over time due to ripening. Thus, we studied the possible influence of the ripening and the batch-to-batch reproducibility of AHG properties. This influence was investigated by changes of the analyte concentration after its interaction with AHG. Pure aqueous solution of CZL with fixed concentration (50 μg/mL) was used to probe the interaction with the portion of AHG. The application of the pure analyte solution enables us to estimate concentration changes using simple absorbance measurements in UV range.

**Figure 3:**
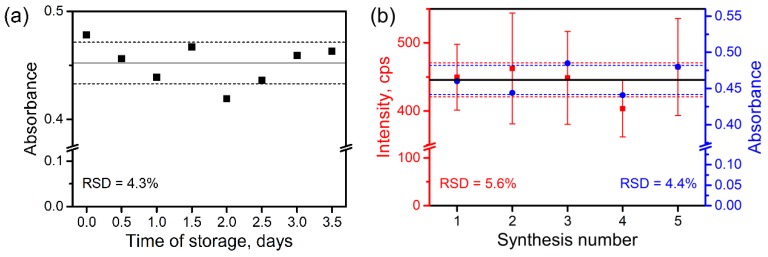
Dependence sorption properties of aluminum hydroxide gel (AHG) on (**a**) time of storage and (**b**) batch-to-batch reproducibility. The results were obtained using absorbance and SERS measurements of aqueous solution of cefazolin (CZL) (50 μg/mL) after its interaction with AHG.

The results show (Figure 3) that the storage time and the batch-to-batch reproducibility have nearly the same deviation around 4%. The analysis of profiles of absorbance spectra (Figure S3a,c) shows that the deviation of the signal is not homogeneous across the whole spectrum. This fact can be explained by significant contribution of Rayleigh scattering of residuary AHG particles. Thus, the deviation of sorption properties should be even lower than found. The batch-to-batch reproducibility was also checked by SERS measurements and the results show satisfactory RSD value of about 6% (Figure 3b and Figure S3b). In the case of SERS, both in- and inter-batch signal deviations also experience influence of inhomogeneous Raman enhancement as the results of deviation of AgNPs size and their aggregates. Therefore, we can conclude that the sorption properties of AHG are satisfactorily reproduced well and stable in time, making AHG suitable for application in analysis.

### 3.3. Influence of the Pretreatment on Analyte Concentration

Because SERS signal after pretreatment by AHG is produced by remained analyte molecules, we estimated possible changes of analyte concentration caused by the pretreatment. Two important side processes can be highlighted: the dilution of analyte and its partial sorption by AHG. The complete removal of water from AHG after preparation is difficult and may lead to speeding-up the process of AHG ripening; i.e., the final AHG portions were prepared as suspension with some excess of water content. Thus, the analyte dilution cannot be avoided during sample pretreatment by AHG. The second process is of particular importance because it leads to the loosing of the analyte molecules making them unavailable for further SERS detection. Unfortunately, precise quantification of the contributions of the both processes is quite a complex task. Therefore, we estimated their total effect measuring the final analyte concentrations after interaction with AHG. The estimations were performed using absorbance measurements in UV range; absorbance bands of all analytes are below 350 nm (Figure S4). The aqueous solutions of pure analytes were used in this study. Two concentrations were used for each analyte to account possible saturation effects within the range of interest.

The results show that the analytes demonstrate different interaction degree with AHG (Table 1). The maximal interaction was observed between CTR: only 1.7% of the analyte molecules remain in the supernatant after interaction with AHG. The concentrations of the other analytes experience 80‒85% reduction after pretreatment. Fortunately, the sensitivity of SERS enables us to overwhelm the decrease of the analyte concentrations and perform quantitative analysis.

### 3.4. SERS Analysis of Spiked Urine and Analytical Performance

#### 3.4.1. Analysis Protocol

The final analysis protocol includes following steps: (i) purification of the urine aliquot using the portion of AHG; (ii) sampling the purified analyte solution and its pH adjusting; and (iii) addition of AgNPs and further SERS detection. Importantly, the second step (sampling) requires taking of the different volumes of the purified analyte solutions depending on the analyte nature: 100 μL for CZL, CPR, and CTX, and 175 μL for CTR and CRX. The increasing of the solution volumes in the case of CTR and CRX is necessary because lower volumes do not enable sensitive detection of these analytes within required range of concentrations. Although this moment reduces the universality of the final protocol, it is not a problem for the practical implementation because TDM implies availability of the information about administrated antibiotics. Lastly, the protocol requires around 15 min to complete.

According to pharmacokinetic measurements [24,25,26,27,28], the concentrations of the used antibiotics are decreased fast from high to low values in the urine of people with normal renal functions. For example, the maximal concentration of CZL in this case can reach 700 μg/mL and then drops down to 30 μg/mL within 24 h after intramuscular injection of 500 mg. On the other hand, the content of the antibiotics in the urine of people with renal failures remains at level of low/moderate concentrations, but for longer time indicating the retention of drug in body and enhancement of possible side effects. In this case the concentration of CZL will remain at the level of 50‒100 μg/mL for several days. Accounting that the selected antibiotics in the most cases have quite similar excretion degree, we used 50‒500 μg/mL range of concentrations as the approximate range for further SERS detection.

#### 3.4.2. Calibration Curves and Influence of Sample Pretreatment

**Figure 4:**
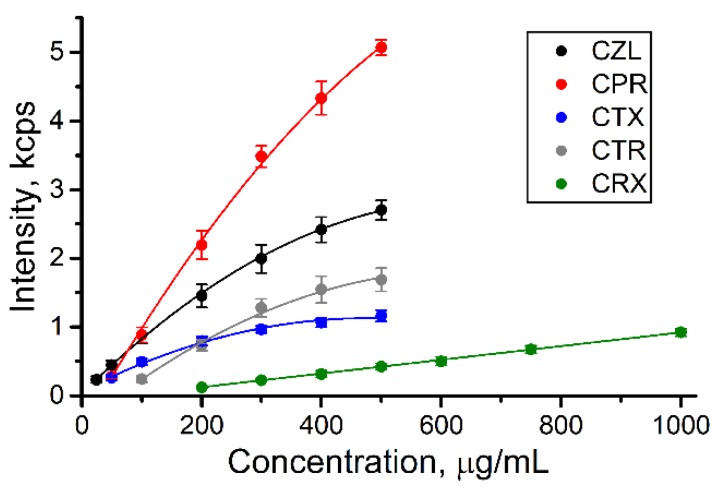
Calibration curves for pretreated urine samples spiked with different cephalosporins. The signal deviation represents reproducibility of analysis for five samples with the same concentrations.

The calibration curves for the urine samples spiked by the antibiotics are shown in Figure 4 (full spectra are provided in Figure S5). The results show that almost all antibiotics can be detected within the concentrations of interest for TDM (50‒500 μg/mL). The signal of CRX was detected at larger concentrations making the protocol suitable for qualitative detection only: the presence of any CRX signal shows the normal analyte excretion while the lack of the signal can indicate the renal failure.

The curves for most antibiotics have nonlinear shape approximated by polynomial functions, while it is linear for CRX (Table S3). Because SERS analysis implies adsorption of the analyte on the surface of SERS substrate, the calibration curve approximately reflects the isotherm of adsorption [29], i.e., dependence of surface coverage on the concentration of adsorbed molecule at constant temperature. The surface coverage depends on the concentration linearly only at low coverage degrees (Henry’s adsorption isotherm) resulted from low analyte concentrations and/or weak adsorption. This fact explains the nonlinear shape of the curves for CZL–CTR (moderate surface coverage) and linear shape of the calibration plot for CRX (low surface coverage). The retention of the analytes during urine pretreatment by AHG (Section 3.3) also depends on the analyte concentration nonlinearly contributing to the final calibration curves. However, all curves are reproducible, and that evidences the applicability and reliability of the protocol for quantification within the required ranges of the concentrations.

The interaction between the analyte and the surface of SERS substrate is the most influencing factor which causes variation of SERS intensity from analyte to analyte and determines the analysis outcomes. The analyte with strong interaction demonstrates the larger response of SERS signal to the increase of the analyte concentration compared to the analyte with weak interaction. Thus, the approximate estimation of the interaction degree for the studied analytes can be performed by analysis of the slopes of the calibration curves (Table S3). According to this analysis, CPR and CRX possess the strongest and weakest interactions with SERS substrate, respectively. CZL and CTR possess quite similar interaction strength and CTX is somewhere between CZL/CTR and CRX. Therefore, we expect that the detection of CPR, CZL, and CTR will be minimally influenced by admixtures such as other drugs or unexpectedly high concentrations of intrinsic urine components. On the other hand, CRX may experience strong negative influence of the admixtures leading to low analysis reliability.

In addition to the interaction between the analyte and SERS substrate, the pretreatment protocol also influences SERS detection. This influence was estimated by comparison with calibration plots for SERS signal of pure aqueous solutions of the analytes (Figures S6 and S7). The linear ranges in the case of pure analytes are very narrow and below the concentrations which have to be detected in urine; larger concentrations lead to signal saturation and were not studied. The analysis of slopes of these plots (Table S3) also shows that CRX possesses low affinity to SERS substrate compare to other analytes. Additionally, we can conclude that the reduction of the analyte concentration during the urine pretreatment (discussed in the previous section) plays a positive role leading to minimization of signal saturation and linearization of the calibration curves. Namely this effect enables us to extend the detection within whole required range of analyte concentrations.

#### 3.4.3. Limits of Quantification and Signal Deviation

From our point of view, the applicability of the limit of detection (S/N = 3 or 3σ) as the analytical characteristic is limited in the case of label-free SERS detection. The adsorption of analyte onto the surface of SERS substrate is an equilibrium process which can be suppressed by competitive interactions between the surface and intrinsic urine components. Importantly, the suppression is not a constant and grows with decreasing the analyte concentration, particularly if the adsorption constant is small. Therefore, calculated limit of detection may not reflect experimentally detectable concentrations and we prefer to use limit of quantification (LOQ; S/N = 10 or 10σ) that reflects the values which are really possible to detect using SERS.

Although the calibration curves are described by polynomial equations (Figure 4; Table S3), the LOQ values were calculated using linear approximations of the ranges of low analyte concentrations. This approach was used because at low concentrations the analyte adsorption is described by linear function (Henry’s law). LOQ values for almost all analytes (except of CRX) are below the concentrations interesting for TDM [24,25,26,27,28] (Table 2) that proves appropriate reliability of the analysis protocol.

The analysis of random error (relative standard deviation—RSD) shows that it does not exceed values normal for SERS analysis: ~15‒20% at the level of minimal studied concentration for each analyte and ~5% at larger concentrations. The urine matrix also contributes to these values increasing intensity of noise signal two times compared to pure analyte solutions (Table S3). Importantly, SERS detection in this study was performed using portable Raman spectrometer in standard cuvette and using low aperture Raman probe (0.22 NA). The comparison with confocal optical systems, which we used earlier [12], demonstrates that the RSD value is reduced from 30 to 15‒20% in the case of portable spectrometer. We explain this by reduction of spatial resolution of the optical system making it less sensitive to low homogeneity of the SERS substrate (suspension of aggregated AgNPs) and providing the averaged SERS signal within the analyzed solution. This result shows an additional advantage (besides of portability) of the portable Raman spectrometers compare to complex spectrometers combined with microscopes.

#### 3.4.4. Limitations and Possible Modifications of the Protocol

As any analysis protocol, the protocol described here also has a list of limitations. First, the protocol does not allow for detection in the morning urine because of too large content of intrinsic components which produce background signal with inappropriate intensity. The analytes with low affinity to the surface of SERS substrate are in particularly influenced by this factor (e.g., CRX). Second, the analytes of interest must be stable and SERS-active in acidic media. Third, AHG is amphoteric that does not allow its usage for purification of the analytes in strong acidic or alkaline solutions. This limitation is not an issue for urinalysis because native urine does not possess such pH values. However, in the case someone wants to adapt the protocol for analysis of the other objects, this feature of AHG should be accounted. Despite the listed limitations, the protocol can be easily corrected for the detection of the other analytes by optimizing (i) the ratio of AHG to urine; (ii) volume of purified urine solution used for SERS detection; (iii) pH adjusting; and (iv) the ratio of pretreated urine to AgNPs.

## 4. Conclusions

Therefore, the main result of the study is the SERS-based analysis protocol, which allows for detection of cephalosporin antibiotics in the real human urine. The potential of the protocol for quantitative detection was shown for four antibiotics. For one antibiotic (cefuroxime), the protocol is applicable only for qualitative analysis because its calibration plot covers only high concentrations possible in the real urine. Therefore, the different excretion of the antibiotics and intensity of their SERS signals restricts universality of the protocol. However, all calibration curves are reproducible and appropriate levels of RSD and LOQ show the high potential and reliability of the protocol for practical application in therapeutic drug monitoring.

## Figures and Tables

**Table 1:** The influence of sample pretreatment by AHG on the concentrations of the analytes. The table represents the maximum of analyte absorption band (λ) (full spectra are in Figure S4) and corresponding calibration equation obtained for pure analyte solutions; concentrations of analyte solution before (C_initial_) and after (C_final_) pretreatment; the content of the analyte in the solution after pretreatment relative to the analyte content before pretreatment (%).

Analyte	λ, nm	Calibration Equation	C_initial_, μg/mL	C_final_, μg/mL	Content, %
CZL	273	y = 0.019x + 0.004 R^2^ = 0.998	100	20	20
			200	32	16
CPR	227	y = 0.033x + 0.015 R^2^ = 0.999	100	19	19
			200	35	18
CTX	232	y = 0.028x − 0.087 R^2^ = 0.999	100	17	17
			200	26	13
CTR	239	y = 0.043x + 0.040 R^2^ = 0.999	100	1.7	1.7
			200	3.4	1.7
CRX	280	y = 0.031x + 0.088 R^2^ = 0.994	100	16	16
			200	31	16

**Table 2:** The summary table of figures of merit for the analysis protocol: the volumes of the analyzed solution used for the SERS analysis after pretreatment, the ranges of concentrations for which the calibration curves were obtained, the limits of quantification achieved during detection of the analytes in urine (LOQ) and pure water (LOQ_water_).

Analyte	Volume, μL	Range, μg/mL	LOQ, μg/mL	LOQ_water_, * μg/mL
CZL	100	25–500	19	1.7
CPR	100	50–500	45	4.1
CTX	100	50–500	34	2.0
CTR	175	100–500	92	1.0
CRX	175	200–1000	280	13

* The volume of the analyte solution used for SERS detection was fixed at 100 μL.

## Supplementary Materials

The following are available online at https://www.mdpi.com/2079-6374/9/3/91/s1. Figure S1: SERS spectra of (**a**) urea (1000 μg/mL) and (**b**) creatinine (2 μg/mL) aqueous solutions at different pH values, Figure S2: An influence of urine dilution on SERS signals of the pure urine with acidic pH (1.5), Figure S3: (**a**) Absorbance spectrum of CZL obtained during the study of temporal stability of AHG. (**b**,**c**) SERS and absorbance spectra of CZL obtained during the study of batch-to-batch reproducibility of AHG synthesis, Figure S4: Profiles of absorbance spectra of the analytes (50 μg/mL), Figure S5: SERS spectra of the spiked urine samples after pretreatment with AHG and pH adjusting to 1.5, Figure S6: SERS spectra of the pure analytes solutions, Figure S7: Linear parts of the calibration curves obtained using registered SERS spectra of the pure analyte solutions (Figure S6), Table S1: Chemical structures of the analytes (cephalosporins), Table S2: Description of the Raman spectrometer used in the study, Table S3: The equations of calibration curves/plots obtained by registration of SERS signals of analyte solutions in pure water and the urine sample spiked by the analytes.
