# Supplementary material for: Application of Aluminum Hydroxide for Improvement of Label-Free SERS Detection of Some Cephalosporin Antibiotics in Urine
Source: Biosensors (Basel). 2019 Jul 23;9(3):91. doi: 10.3390/bios9030091 (PMC6784367; doi:10.3390/bios9030091)
Supplement: Supplementary file 1 [file biosensors-09-00091-s001.pdf]

Supplementary Material

Application of Aluminum Hydroxide for Improvement of Label-Free SERS Detection of Some Cephalosporin Antibiotics in Urine

Natalia E. Markina, Alexey V. Markin*

Saratov State University, Astrakhanskaya 83, 410012 Saratov, Russia
* Correspondence: av_markin@mail.ru

Table 1. Chemical structures of the analytes (cephalosporins):.

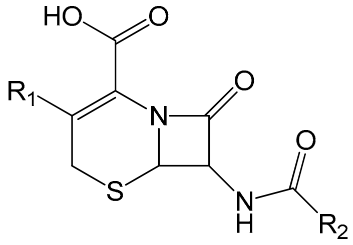

Analyte	R_1 –	– R_2	Generation	Molecular weight, g/mol
CZL	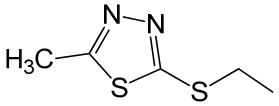	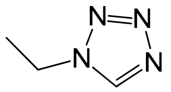	1	454.5
CRX	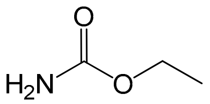	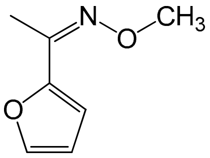	2	424
CPR	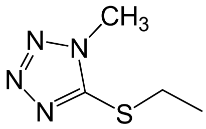	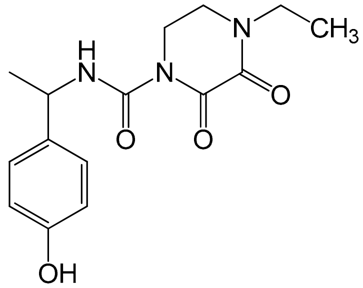	3	646
CTX	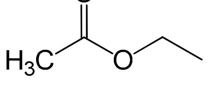	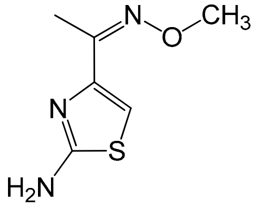	3	455.5

Biosensors **2019**, *9*, 91; doi:10.3390/bios9030091

CTR	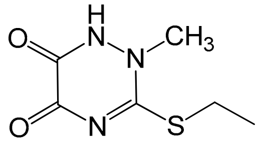	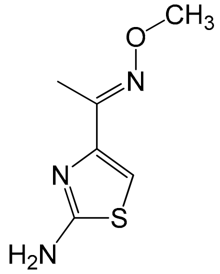	3	554.5

Table S2. Description of the Raman spectrometer used in the study.

Detail	Description
Spectrometer	QEPro
Diffraction gratings	1200 g/mm (Grating H35)
Raman shift range	150–3100 cm^{-1}
Slit	100 μm
Laser	638 nm<br>18 mW (power at the sample)<br>(Laser-638-LAB-FCA)
Raman probe	FC excitation and SMA 905 collection<br>(RIP-RPB-638-SMA-SMA)
Holder	Universal for Raman, fluorescence,<br>and absorbance measurements (OOA-HOLDER-RFA)
Software	OceanView

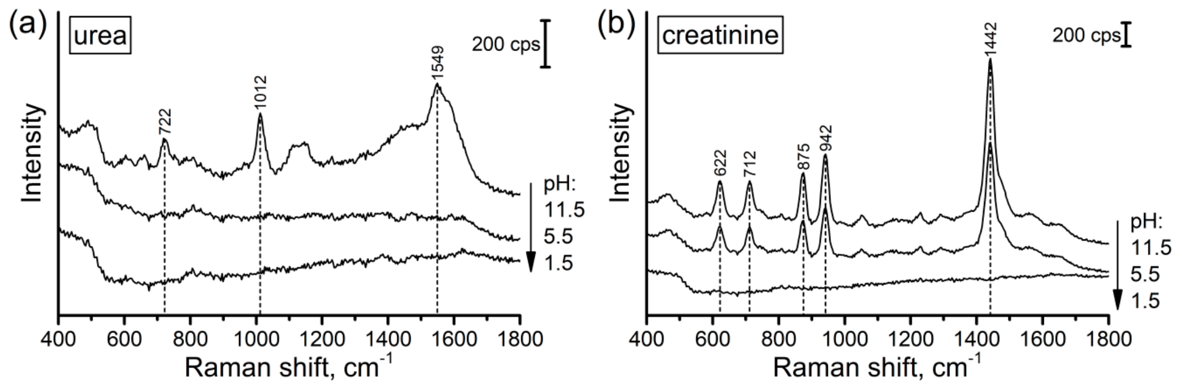

Figure S1. SERS spectra of (**a**) urea (1000 μg/mL) and (**b**) creatinine (2 μg/mL) aqueous solutions at different pH values.

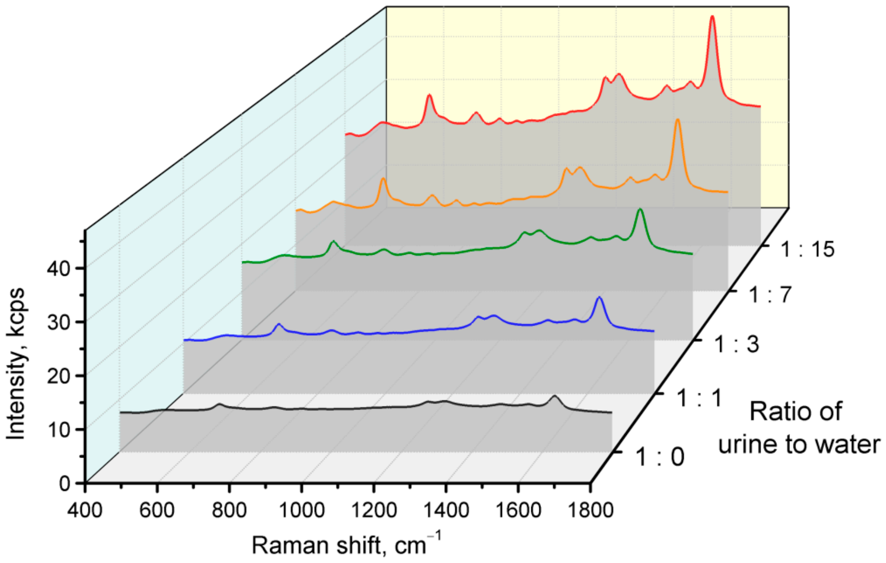

Figure S2. An influence of urine dilution on SERS signals of the pure urine with acidic pH (1.5).

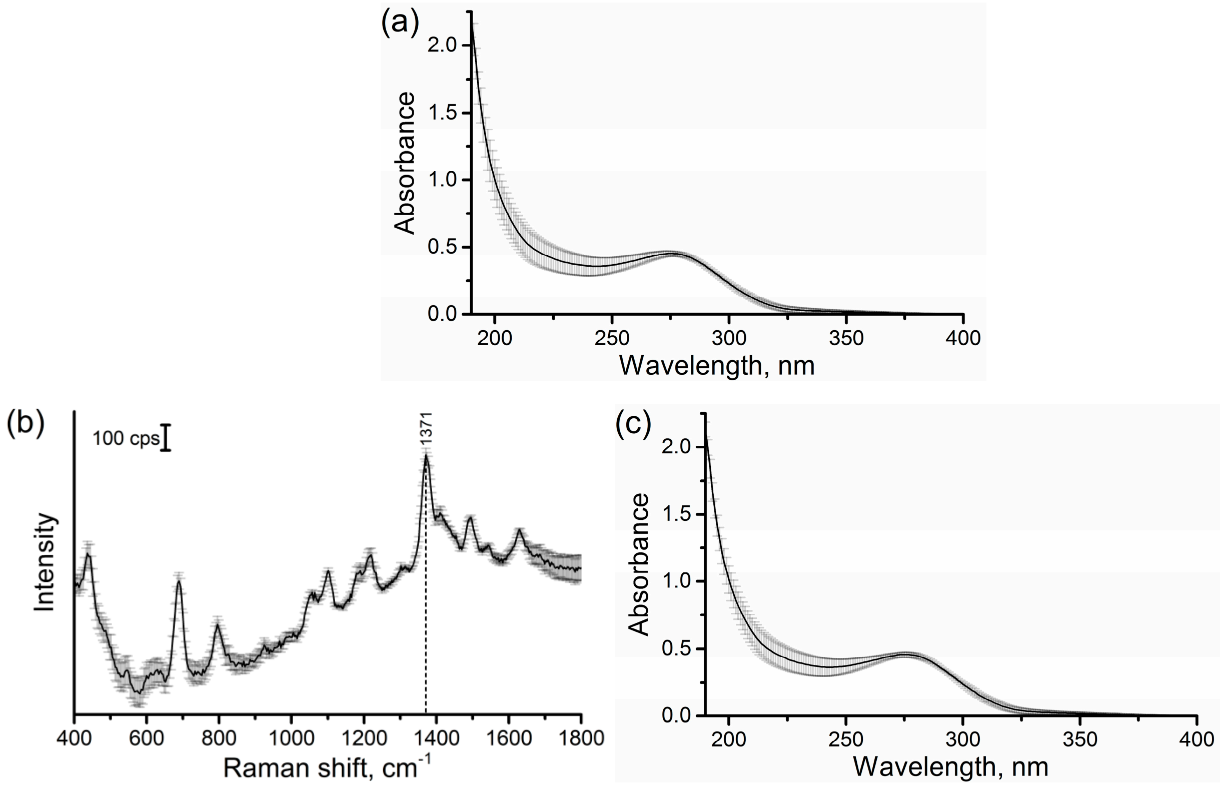

Figure S3. (**a**) Absorbance spectrum of CZL obtained during the study of temporal stability of AHG. (**b**,**c**) SERS and absorbance spectra of CZL obtained during the study of batch-to-batch reproducibility of AHG synthesis.

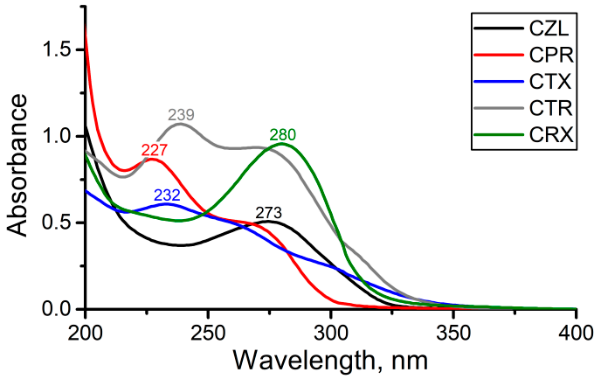

Figure S4. Profiles of absorbance spectra of the analytes (50 μg/mL)

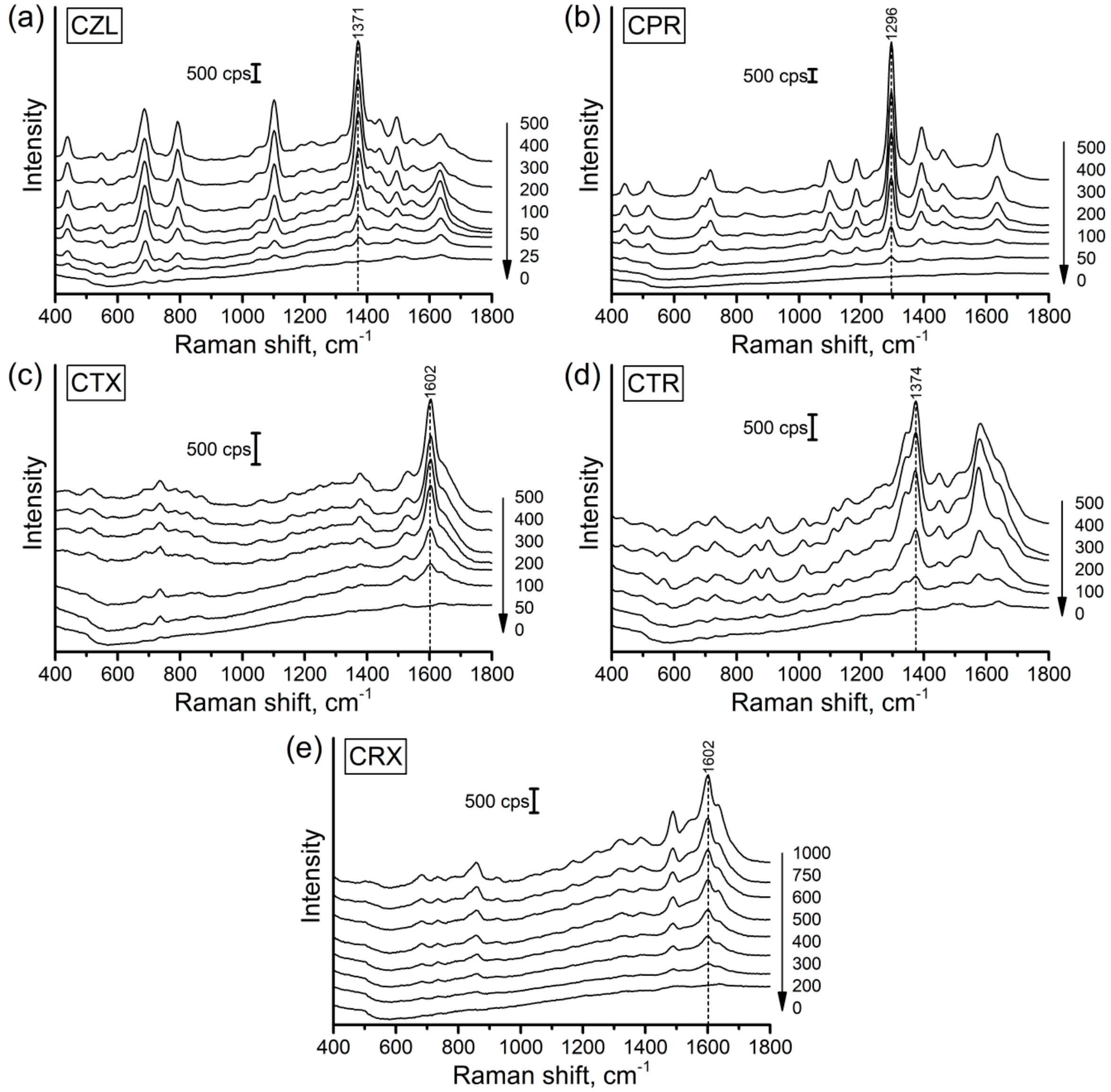

Figure S5. SERS spectra of the spiked urine samples after pretreatment with AHG and pH adjusting to 1.5. Marked bands were used to plot calibration curves (Figure 4 in the main text).

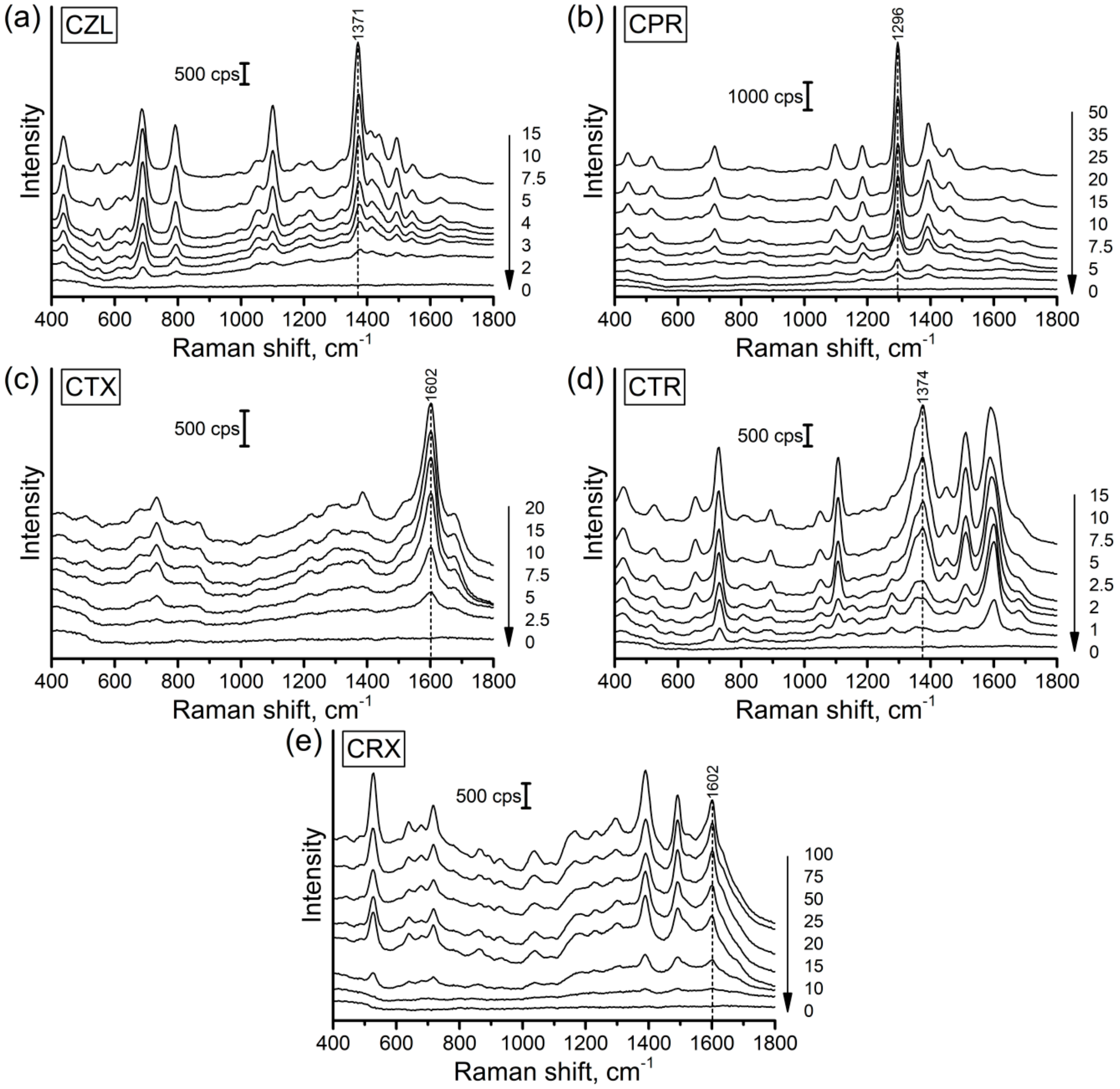

Figure S6. SERS spectra of the pure analytes solutions. Marked bands were used for plotting calibration curves (Figure S7).

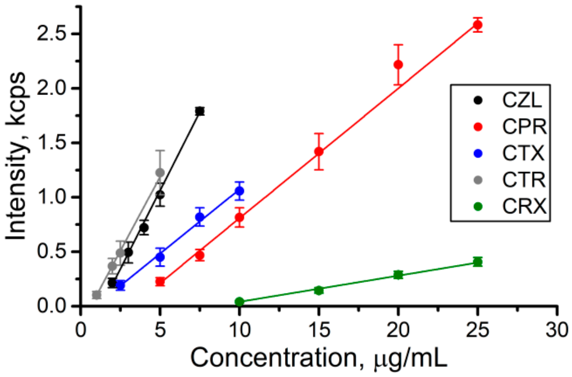

Figure S7. Linear parts of the calibration curves obtained using registered SERS spectra of the pure analyte solutions (Figure S6).

Table S3. The equations of calibration curves/plots obtained by registration of SERS signals of analyte solutions in pure water and the urine sample spiked by the analytes. I_{SERS}, C, and σ are the intensity of SERS signal, the analyte concentration, and the level of background signal, respectively. The intensity of SERS and background signals is measured in counts per second (cps).

Analyte	Pure water	σ	Spiked urine		
			Whole range	Linear range	σ
CZL	$I_{SERS} = 288\ C - 378$<br>$R^2 = 0.999$	10	$I_{SERS} = -\ 0.007\ C^2 + 8.6\ C + 30$<br>$R^2 = 0.999$	$I_{SERS} = 7.4\ C + 66$<br>$R^2 = 0.994$	20
CPR	$I_{SERS} = 119\ C - 385$<br>$R^2 = 0.992$	10	$I_{SERS} = -\ 0.009\ C^2 + 15\ C - 479$<br>$R^2 = 0.999$	$I_{SERS} = 13\ C - 362$<br>$R^2 = 0.999$	22
CTX	$I_{SERS} = 118\ C - 107$<br>$R^2 = 0.996$	13	$I_{SERS} = -\ 0.005\ C^2 + 4.4\ C + 68$<br>$R^2 = 0.994$	$I_{SERS} = 3.6\ C + 97$<br>$R^2 = 0.991$	22
CTR	$I_{SERS} = 272\ C - 171$<br>$R^2 = 0.998$	11	$I_{SERS} = -\ 0.006\ C^2 + 7.0\ C - 409$<br>$R^2 = 0.995$	$I_{SERS} = 5.0\ C - 266$<br>$R^2 = 0.999$	20
CRX	$I_{SERS} = 24.0\ C - 199$<br>$R^2 = 0.996$	11	$I_{SERS} = 0.99\ C - 74$<br>$R^2 = 0.999$	$I_{SERS} = 1.0\ C - 74$<br>$R^2 = 0.999$	20
